# Sociocultural adaptation, translation and pre-testing of the Kannada version of Vanderbilt Head and Neck Symptom Survey 2.0

**DOI:** 10.1186/s41687-022-00523-1

**Published:** 2022-11-25

**Authors:** Naina Johnson, Janet Jaison Varghese, Krishna Sharan, Venkataraja U. Aithal, Barbara Murphy

**Affiliations:** 1 Department of Speech and Hearing, Manipal College of Health Professions, MAHE, Manipal, Karnataka India; 2 Department of Radiotherapy and Oncology, Kasturba Medical College, MAHE, Manipal, Karnataka India; 3 Vanderbilt University Medical Centre, Nashville, TN USA

**Keywords:** VHNSS 2.0, PROMs, Head and neck cancer, Quality of life

## Abstract

**Background:**

Head and neck cancer (HNC) patients often undergo radiation therapy as part of their treatment. However, radiation therapy can have many side effects, including oral toxicities. Evaluating these toxicities is often considered a challenging task for practicing clinicians due to the lack of assessment tools. The objective of this study is to culturally adapt, translate and validate the Vanderbilt Head and Neck Symptom Survey (VHNSS), an instrument designed to assess oral toxicities and changes in oral functioning in HNC patients receiving radiation therapy.

**Methods:**

The VHNSS 2.0 was first culturally adapted and translated, following which 36 HNC patients undergoing radiation therapy were identified through the incidental sampling method. The translated version of VHNSS 2.0 was then administered to these patients. Internal consistency was assessed using Cronbach’s alpha and Mc Donald’s Omega. Test–retest reliability was also analyzed.

**Results:**

Items of the translated version of VHNSS 2.0 showed good content validity. The omega values yielded higher reliability coefficients than the Cronbach’s alpha coefficient. Test–retest reliability was found to be 0.8, indicating good reliability.

**Conclusions:**

Results of this study suggest that the translated Kannada version of the VHNSS 2.0 is linguistically equivalent to the original version. Hence, this tool can be considered a valid and reliable patient-reported tool to evaluate oral symptomatology in HNC patients speaking the Kannada language.

## Background

The term head and neck cancer (HNC) refers to the malignancies that usually arise from the squamous cells that line the mucosal surfaces of the head and neck. Head and neck squamous cell carcinomas (HNSCCs) are the most frequent histological subtype, accounting for up to 90% of HNCs. It is the sixth most frequent cancer worldwide, annually accounting for more than 550,000 cases and about 300,000 fatalities every year [1, 2]. Tongue and buccal mucosa cancers are common in India and have been linked to the traditional practice of chewing paan, betel leaf, and tobacco [3].

HNSCCs are most frequently seen as locally advanced tumours, causing significant symptoms in a patient. The etiology for HNC can be manifold. It may be related to certain environmental factors and lifestyle patterns. The two most important risk factors for HNCs, particularly cancers of the oral cavity, hypopharynx, and larynx, are reported to be alcohol and tobacco use [4, 5]. The risk of developing cancer is higher in persons who consume tobacco and alcohol rather than those who consume either of these two [6, 7]. Infection with cancer-causing strains of the human papillomavirus (HPV), particularly HPV type 16, is linked to oropharyngeal tumours of the tonsils and base of the tongue [8, 9]. The use of paan (betel quid) in the mouth, a common custom in Southeast Asia, has been linked to a higher risk of oral cancer [10, 11]. Other causes may include Epstein-Barr virus infection, occupational exposure, radiation exposure, or underlying genetic conditions [12–15].

The major treatment choices available for HNC are surgery, chemotherapy (CT), and radiation therapy (RT). Most often, these approaches are used in combination for treatment. When the treatment comprises of surgery as the primary mode, followed by post operative radiation therapy, this type of RT is referred as adjuvant radiation therapy.

The primary goal of HNC treatment is to keep the disease under control while preserving as much function as possible. The popularity of organ preservation regimes in cancer care has increased the use of non-surgical treatment options, namely radiation therapy in combination with chemotherapy. This form of RT is called as definitive radiation therapy.

During RT, toxicities develop due to the disruption of healthy cells near the treatment site, especially in regions receiving a high dose. The severity and type of toxicity experienced by each person may vary considerably as these depend on the treatment sites. Radiation therapy reactions sometimes occur in the second or third week following the commencement of treatment and can often continue for several weeks after treatment is completed.

Radiation therapy for HNC may present several acute toxicities like dry mouth, sore mouth and gums, swallowing difficulties, stiffness in the jaw, nausea, hair loss, lymphedema, tooth decay, etc. Patients may also develop redness, irritation, thicker saliva, ear pain, nausea during and after treatment, as well as a loss of taste, which can reduce appetite and impact nutrition. Patients may experience jaw stiffness and may be unable to open their mouths as wide as they could. In many patients, these adverse effects will fade over time, but long-term complications such as swallowing difficulties are known to persist.

Symptoms such as fatigue, nausea, or pain can only be identified and described appropriately by the person experiencing it. It is a subjective indicator of disease or any physical distress. A sign, on the other hand, is any objective evidence of disease that can be recognized by the patient or treating professionals. Evaluating these symptoms that arise as a result of radiation, is often considered a challenging task for practicing clinicians. It is often difficult for the patients to describe the magnitude of the symptoms and its effect on their day-to-day life.

Patient-reported outcome measures (PROMs) provide information on different areas of a patient’s health status that are important to their quality of life (QOL) such as functionality as well as an integration of physical, mental, and social health. PROM tools can be generic, i.e., applied across different populations, or condition-specific, where they are specific or unique to particular diseases or sectors of care. Patient-reported outcomes are critical for determining whether healthcare interventions and procedures improve a patient’s health and QOL. PROMs can be used by clinicians to improve patient-provider communication and patient involvement in decision-making. “The Vanderbilt Head and Neck Symptom Survey (VHNSS)” is one such PROM tool that can be used in the HNC population.

The VHNSS is an instrument designed specifically for HNC patients to assess oral toxicities and changes in oral functioning in patients with HNC who receive radiotherapy. It was first developed by Murphy et al. in the year 2009 to provide a more comprehensive assessment of oral health problems in the HNC population of the United States [16]. However, the initial version did not account for certain important domains like mucosal sensitivity and dental health. This led to the development of another revised version, i.e., VHNSS 2.0.

This version consists of 10 domains along with 3 single items: making it a total of a 50-item questionnaire. The domains are related to the patient’s nutrition, swallowing solids, swallowing liquids, dry mouth, mouth pain, general pain, mucus, voice/communication, hearing, taste/smell, teeth, neck range of motion, and trismus. Scoring is done on an 11-point Likert scale.

Few of the tools that are used clinically across the world to assess the symptoms and toxicities in HNC patients include the Functional Assessment of Cancer Therapy (FACT), MD Anderson Dysphagia Inventory (MDADI), Sydney Swallow Questionnaire (SSQ), Head and Neck Patient Symptom Checklist (HNSC) and the Eating Assessment Tool (EAT 10). In India, regional patients are often unfamiliar with the English language and there is a dearth of patient-reported tools in Kannada that address oral deficits in HNC patients. Questionnaires that have been already translated and validated into the Kannada language include the Dysphagia Handicap Index (DHI) and the EAT-10. The DHI is a questionnaire with 25 items, which evaluates a patient’s overall or general quality of life. It consists of 30 items in total and is divided into three main domains with 10 items each: physical, functional, and emotional. EAT-10 is yet another self-administered tool in Kannada for the population with dysphagia. This tool has a total of 10 questions related to the general quality of life but does do not address the oral toxicities that arise due to treatment for HNC.

In this study, a sociocultural adaptation and translation of the VHNSS 2.0 in Kannada has been done and it was clinically validated on 36 HNC patients undergoing chemoradiation. The internal reliability was assessed using Cronbach’s alpha coefficient. As the use of alpha coefficient has been critiqued by psychometricians stating its underestimation of reliability [17], we calculated the Mc Donald’s omega coefficient also. Test–retest reliability was also assessed.

## Method

### Ethical considerations

Permission to translate and use the questionnaire was obtained from Dr. Barbara A. Murphy, the original author of the VHNSS 2.0. The research committee and ethics committee of the institution approved the study (IEC no: 458/2021). This study has also been registered under the Clinical Trial Registry of India (CTRI reg no: CTRI/2021/10/037624). Informed consent was obtained from each participant before collecting data.

### Procedure

**Fig. 1:**
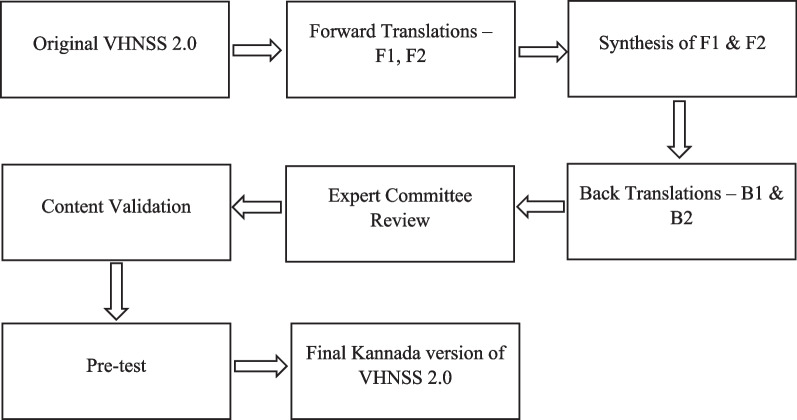
Flowchart for the translation process of the VHNSS 2.0 in Kannada

The study was carried out in two phases. The first phase involved socio-cultural adaptation, translation, and content validation of the original VHNSS 2.0 to the Kannada-speaking community. In the second phase, the translated VHNSS 2.0 questionnaire was clinically validated among HNC patients. A flowchart of the process is depicted in Fig. 1.

## Phase 1—Socio-cultural adaptation, translation, and content validation of the VHNSS 2.0 to Kannada speaking community

### Sociocultural adaptation

Before the process of translation, the original questionnaire was read thoroughly, and the sociocultural appropriateness of the items listed in the questionnaire was analyzed by two English-Kannada bilinguals with an undergraduate degree. It was also ensured that they were not health professionals in order to reduce the risk of overseeing medical language. They aimed to informally evaluate that the items of the instrument are equally relevant and acceptable in the target population (Kannada speaking community) just as they are in the original population (English speaking community). The sociocultural appropriateness was assessed in terms of the relevance of the contents of the questionnaire in the context of Indian culture and the understandability of the words used in the questionnaire among the patient population with minimum educational qualifications.

### Translation and Content validation

#### Forward translation (F1, F2)

The original VHNSS 2.0 questionnaire was independently translated into two Kannada versions of the same (F1, F2) by two translators who were native Kannada speakers and proficient in English. Both the translators were teachers by profession. The translators were informed about the areas assessed in the questionnaire to provide a relevant translation from a clinical perspective. After the two forward-translated versions were completed, one of the translators and a third person were involved in synthesizing the result of the forward translations.

#### Backward translation (B1, B2)

Two Kannada-English bilinguals from non-medical backgrounds then translated the synthesized forward translated version back into English. This served as a check for validity to know if the translated version and original version reflect each other in terms of content. Both translators had adequate proficiency in both English and Kannada. They were not given a reference to the original VHNSS 2.0 version.

#### Expert committee review

An expert committee consisting of a five-expert panel (including an HNC specialist, speech-language pathologist, health inspector and two translators) reviewed the items of the Kannada version and compared them to the original version. These experts were English-Kannada bilinguals with significant experience with HNC (except for the two translators). The translators hailed from the north and south regions of Karnataka and their inclusion in this committee was mainly to check for any cultural discrepancies in words or items that may arise owing to the regional differences in the Kannada language. This committee's task was to merge all the versions and create the pre-final version of the questionnaire that would be used for the field test. Analysis of all the translations was carried out and inconsistencies were checked. The original questionnaire and each translated version (forward and backward) were included in the material at the disposal of the committee.

The committee ensured that equivalence was reached between the original and translated versions in mainly 4 areas:

1. Semantic Equivalence –Words that can have multiple meanings were discussed and the appropriate word was chosen. Grammatical discrepancies were resolved.
2. Idiomatic Equivalence—Equivalent expressions for hard to translate idioms and colloquialisms were devised.
3. Experiential Equivalence—Tasks or questions that are not practiced in the target country or culture were replaced with similar items present in the targeted culture.
4. Conceptual Equivalence—Words that meant different things conceptually between cultures were avoided.

Content validation of the translated questionnaire was done with the help of five postgraduate SLPs. They were asked to evaluate the items of the translated version of the questionnaire on a five-point Likert scale (0–4) in terms of ambiguity, cultural appropriateness, and clarity. Changes were incorporated into the final version of the questionnaire.

## Phase 2—Pre-testing of the translated questionnaire in the HNC patient population

The current study was time bound; therefore, we chose to recruit participants using an incidental sampling method. 36 participants from the target population i.e., HNC patients undergoing radiation therapy, were recruited to administer the final version of the questionnaire. The sample size was calculated using the formula N = 4PQ/d^2^, where N is the sample size calculated, P is the prevalence (90% or 0.9), Q = 1−P, and d is the precision or allowable error (10%).

Participants in the study were diagnosed with HNC (oral, nasopharyngeal, oropharyngeal, laryngeal, or hypo laryngeal, lingual) and were between 18 and 80 years of age. The participants were on definitive or adjuvant radiation therapy at the time of enrollment in the study and had a minimum educational qualification of 7th grade. Patients with any cognitive, neurological, or psychological impairment, and those receiving palliative radiation therapy were excluded from the study. Voluntary participation was ensured, and the recruited participants signed consent forms before taking part in the study.

The participants were asked to self-administer the tool and if they had any clarifications or doubts, the researcher would clarify the same. On completion of the questionnaire, 10 participants were randomly selected and requested to participate in a face-to-face interview to discuss the questionnaire in terms of clarity and understanding. The researchers documented the input and utilized it to further revise the translated version of the questionnaire.

For test–retest reliability, 10 participants from the target population were asked to fill the VHNSS tool again within a week.

### Statistical analysis

Content Validity Index (CVI) value for evaluating the questionnaire's content validity was determined using the Microsoft Excel software. A CVI value = 1 was considered acceptable [18, 19]. Statistical data analysis was carried out using Jamovi version 2.3.18.0 (https://www.jamovi.org, Sydney, Australia). Internal reliability was assessed using Cronbach’s alpha coefficient and Mc Donald’s omega coefficient.

As per Cronbach and Shavelson (2004), Cronbach’s alpha α: ≥ 0.9 is excellent, values α: ≥ 0.8 < 0.9 is good, α: ≥ 0.7 < 0.8 is acceptable, α: 0.6 < 0.7 is questionable, α: ≥ 0.5 < 0.6 is poor and α: < 0.5 as unacceptable [20].

For test–retest reliability, values less than 0.5 are considered poor, values between 0.5 and 0.75 depict moderate reliability, values between 0.75 and 0.9 indicate good reliability, and values greater than 0.90 are suggestive of excellent reliability [21].

## Results

### Socio-cultural adaptation

The two English-Kannada bilinguals assessed the sociocultural appropriateness of the questionnaire in terms of the relevance of the contents in the context of Indian culture and the understandability of the words used in the questionnaire among the patient population. Both agreed that no items on the questionnaire were culturally irrelevant or inappropriate to the Indian culture.

### Forward translation

The two translators who converted the original English version of the VHNSS 2.0 into two independent Kannada versions (F1, F2), found the items in the questionnaire remarkably easy to translate and did not express any ambiguity regarding words or items.

One of the translators and a third person synthesized the two Kannada versions (F1, F2). A few of the conflicting questions that they encountered and the consensus that they arrived at are described below.

- The first question under the “yes or no” subsection: “I currently have a feeding tube in place.” Usually, ‘feeding tube’ is just referred to as a ‘tube’ or ‘pipe’ colloquially. Usage of the literal translation of the term in the Kannada language is not observed or very rare. The translators provided the term ‘  ’, which translated back to ‘food pipe’ instead of ‘feeding tube’. Following the expert committee review, the term was changed to ‘  ’ which means ‘food providing pipe’. It was agreed that the latter seemed more meaningful.
- Item 18: “I have thick mucous or phlegm.” The terms used to address ‘mucous or phlegm’ were ‘  ’ during the translation process. However, the term ‘  ’ is not frequently used or understood by most patients. So, ‘  ’ was used to refer to both mucous and phlegm.
- Item 3, 4, 34, 39: The word order in these sentences was reversed to make the item more comprehensive in Kannada. For example, the original item “I have less desire to eat due to taste change” became “Since there is a change in taste, there is a reduced desire to eat” after translation.

### Backward translation

The back-translated versions done by two translators were slightly different from the original version. However, the changes were not so significant as to bring about a change in the meaning of the original items. Possible alternatives were also discussed among the experts, changes were incorporated and another modified version of the VHNSS 2.0 Kannada was developed.

### Expert committee review

The committee checked for semantic, idiomatic, experiential and conceptual equivalence between the original and translated items of the questionnaire. Table 1 describes the cultural modifications that were offered by the experts.

**Table 1:** Modifications suggested by the experts and translators for the culturally adapted VHNSS 2.0

Item No/Original item	First version	Second version with cultural modifications	Rationale
“I have lost my appetite.”			More accurate
“I have thick mucus or phlegm.”			More comprehensive and culturally appropriate Experiential equivalence maintained
“My average pain level over the last week has been…”			Better clarity Semantic equivalence maintained
“The average relief from my pain medication is…”			Better clarity Semantic equivalence maintained
“I have a burning sensation in the lining of my mouth and throat.”			Simple and clear Semantic equivalence maintained
“The lining of my mouth and throat is sensitive to dryness.”			Clear and culturally more appropriate Semantic equivalence maintained
“I have limitations in the ability to open or move my jaw.”			More specific and clear Semantic equivalence maintained

### Content validity

Five experts rated all the items in the questionnaire (2 instructions; 3 yes or no questions; 50 items) independently. “Item Level-Content Validity Index” (I-CVI) was 1 for all the items except one. One item had an I-CVI of 0.8 but the item was maintained as it is. The overall “Scale-Level CVI” (S-CVI) was calculated to be 0.99. Both these values suggest that the questionnaire has excellent content validity.

### Testing of the final version

The final Kannada version of the VHNSS 2.0 was administered to thirty-six patients who fulfilled the inclusion criteria. Recruitment of these patients took place between November 2021 to January 2022. All of them participated in the study with consent. Table 2 discusses the characteristics of participants in the study. During the cognitive debriefing process involving 10 randomly selected participants, only 2 patients reported that they had difficulty understanding how to mark the responses. Clarification was offered by the primary investigator and the patients once again attempted to complete the questionnaire. None of the recruited participants had any suggestions or modifications to offer regarding the translated items.

**Table 2:** Characteristics of study participants (n = no. of participants)

	n (%)
Gender	
Men	31 (86.1%)
Women	5 (13.8%)
Age (in years)	
Median (range)	55.5 (36–74)
Education	
Secondary	21 (58.3%)
Higher secondary	11 (30.5%)
Under-graduation	3 (8.3%)
Post-graduation	1 (2.7%)
Primary Tumour Site	
Buccal mucosa	7 (19.4%)
Tongue	6 (16.6%)
Floor of mouth (FOM)	1 (2.7%)
Alveolus	1 (2.7%)
Gingivobuccal sulcus, palate, and maxilla	2 (5.5%)
Oropharynx	5 (13.8%)
Hypopharynx	1 (2.7%)
Pyriform sinus	2 (5.5%)
Larynx	6 (16.6%)
Supra-glottis	3 (8.3%)
Glottis	2 (5.5%)
TNM stage	
I	5 (13.8%)
II	9 (25%)
III	7 (19.4%)
IV	15 (41.6%)
Type of Treatment Modality	
Definitive RT	8 (22.2%)
Definitive CT-RT	10 (27.7%)
Adjuvant RT	12 (33.3%)
Adjuvant CT-RT	6 (16.6%)

Out of the 36 participants, 86.1% (n = 31) of the participants were males and the rest females (n = 5), 58.3% (n = 21) had completed secondary education. Cancer of the buccal mucosa was the most common condition (19.4%), followed by cancer of the tongue and larynx (16.6%). The least common primary tumour sites were the floor of the mouth, alveolus, and hypopharynx (2.7%). Stage IV cancer was most prevalent (41.6%). Out of all 36 participants, 12 (33.3%) received adjuvant RT, 10 (27.7%) received definitive CT-RT, and 8 (22.2%) received definitive RT and 6 (16.6%) received adjuvant CT-RT.

Table 3 depicts the descriptive statistics of the VHNSS.2.0 Kannada version.

**Table 3:** Descriptive statistics of the VHNSS.2.0 Kannada version

Domains with individual items	Possible scores		Observed Scores				
	Minimum	Maximum	Mean	Median	SD	Minimum	Maximum
Nutrition	0	40	16.19	14	10.70	0	40
Q1-Weight loss	0	10	3.12	2.5	3.21	0	10
Q2-Appetite loss	0	10	3.40	3	3.52	0	10
Q3-Supplement use	0	10	5.38	5	3.88	0	10
Q4-Trouble maintaining weight	0	10	4.86	5	3.55	0	10
Swallowing Solids	0	70	23.28	19.5	17.24	0	60
Q5-Trouble eating solids	0	10	5.67	5.5	3.84	0	10
Q7-Food gets stuck in mouth	0	10	2.85	1	3.43	0	10
Q8-Food gets stuck in throat	0	10	1.79	0	3.11	0	9
Q10-Chokes on solids	0	10	2.06	0	3.15	0	10
Q11-Cough after swallow	0	10	3.14	2	3.35	0	10
Q12-Swallowing takes effort	0	10	3.97	3	3.38	0	10
Q13-Eating takes longer	0	10	4.43	5	3.45	0	10
Swallowing Liquids	0	20	4.14	2.5	5.55	0	20
Q6-Trouble drinking liquids	0	10	2.49	0	3.31	0	10
Q9-Chokes on liquids	0	10	1.77	0	3.12	0	10
Dry Mouth	0	40	11.69	9	11.57	0	40
Q14-Dry mouth	0	10	3.25	3	3.32	0	10
Q15-Difficulty chewing	0	10	2.92	2	3.15	0	10
Q16-Difficulty sleeping	0	10	2.46	0	3.13	0	10
Q17-Difficulty speaking	0	10	3.23	2	3.71	0	10
Mucous	0	40	11.31	5.5	12.87	0	40
Q18-Mucous/phlegm	0	10	4.29	5	3.47	0	10
Q19-Choking	0	10	2.14	0	3.29	0	10
Q20-Difficulty swallowing	0	10	2.97	0	3.92	0	10
Q21-Sleep affected	0	10	2.47	0	3.78	0	10
Mouth Pain	0	80	29.53	21.5	23.12	0	80
Q22-Sores cause pain	0	10	3.34	1	3.95	0	10
Q23-Trouble swallowing	0	10	4.51	5	3.71	0	10
Q24-Trouble speaking	0	10	4.11	4	3.65	0	10
Q44-Sensitivity of mouth/throat	0	10	3.63	3	3.52	0	10
Q45-Sensitivity to acidic, spicy or hot foods	0	10	4.75	4.5	3.63	0	10
Q46-Sensitivity to dryness	0	10	3.97	2	4.09	0	10
Q47-Altered food choices	0	10	4.15	3.5	3.80	0	10
Q48-Difficulty brushing teeth	0	10	2.70	0	3.83	0	10
General Pain	0	40	13.97	11	9.54	0	34
Q25-Average pain level	0	10	3.92	3.50	3.02	0	10
Q26-Worst pain level	0	10	3.80	4	2.87	0	10
Q27-Average relief from pain medication	0	10	5.20	5	3.18	0	10
Q28-Pain causing difficulty sleeping	0	10	3.03	1	3.93	0	10
Communication/Voice	0	30	10.61	7.5	10.23	0	30
Q29-Trouble speaking	0	10	4.34	5	4.00	0	10
Q30-Hoarse voice	0	10	3.39	2	3.60	0	10
Q31-Trouble being understood	0	10	3.47	3	3.69	0	10
Hearing (Q32)	0	10	1.77	0	3.53	0	10
Taste and Smell	0	60	22.17	24	16.92	0	60
Q33-Taste altered	0	10	5.34	5	4.03	0	10
Q34-Decreased desire to eat	0	10	5.73	7	3.80	0	10
Q35-Altered food choices	0	10	4.21	3.5	3.80	0	10
Q36-Decreased food eaten	0	10	4.82	5	3.55	0	10
Q37-Sense of smell changed	0	10	2.09	0	3.41	0	10
Q38-Altered food choices	0	10	1.58	0	2.92	0	10
Teeth	0	50	5.88	4	7.39	0	33
Q39-Difficulty chewing	0	10	2.38	0	3.28	0	10
Q40-Teeth sensitive to hot, cold, sweet foods	0	10	2.96	3	3.06	0	8
Q41-Teeth feel looser	0	10	1.59	0	2.86	0	10
Q42-Cracking/chipping teeth	0	10	0.75	0	2.17	0	10
Q43-Trouble with dentures	0	10	1.42	0	2.52	0	9
Jaw (Q49)-Limited mouth opening	0	10	3.14	0.5	3.80	0	10
Neck (Q50) -Limitations in neck/shoulder movement	0	10	2.65	0	3.55	0	10

### Internal consistency

Cronbach’s alpha coefficient and Mc Donald’s omega coefficient were used to calculate the internal consistency of the domains of the questionnaire. Table 4 gives Cronbach’s alpha values and the Mc Donald’s omega values of the domains of the questionnaire. The overall value of α was 0.93 and omega value was calculated to be 0.96, which indicated good internal reliability of the questionnaire. The domain-specific values were as follows—nutrition (α = 0.74; ω = 0.76), swallowing solids (α = 0.88; ω = 0.89), swallowing liquids (α = 0.68; ω = 0.68), dry mouth (α = 0.90; ω = 0.91), mucous (α = 0.93; ω = 0.93), pain in the oral cavity (α = 0.92; ω = 0.92), general pain (α = 0.79; ω = 0.85), speech/communication (α = 0.90; ω = 0.90), smell and taste (α = 0.86; ω = 0.86), teeth (α = 0.87; ω = 0.91). All domains had values greater than 0.70 except two, i.e., swallowing liquids.

**Table 4:** Internal consistency using Cronbach’s alpha coefficient and Mc Donald’s Omega Coefficient

Domains	No. of items	Cronbach's α	McDonald's ω
Nutrition	4	0.74	0.76
Swallowing Solids	7	0.88	0.89
Swallowing Liquids	2	0.68	0.68
Dry Mouth	4	0.90	0.91
Mucous	4	0.93	0.93
Mouth Pain	8	0.92	0.92
Pain	4	0.79	0.85
Speech/Voice	3	0.90	0.90
Taste and Smell	6	0.86	0.86
Teeth	5	0.87	0.91

### Test–retest reliability

Ten participants were chosen at random from the sample group and asked to complete the questionnaire twice, with a one-week gap between each time. The overall test–retest reliability coefficient was found to be 0.8, which indicates good/acceptable reliability.

## Discussion

In the current study, we carried out a sociocultural adaptation, translation, and validation of the original VHNSS 2.0 version to its Kannada version. The results indicate that this Kannada version of the VHNSS 2.0 is a good, reliable, and valid tool to assess oral toxicities in HNC patients undergoing radiation therapy.

Although there are several patient-reported tools available to assess the quality of life in HNC patients, none of them specifically target domains related to oral health and a Kannada version of those tools do not exist. Hence, this study was carried out. The original VHNSS version was initially developed as a tool for screening to assess oral toxicities and changes in oral functioning in HNC patients receiving radiotherapy and to manage those changes. The revised version, i.e., VHNSS 2.0, was more domain-specific with good psychometric properties [22].

During the translation process of the questionnaire—both forward and backward, as well as cross-cultural adaptation, discrepancies were noticed among the experts involved. Certain items had to be grammatically and culturally modified to be culturally acceptable in Kannada and comprehensive. For example, the item “My average pain level over the last week has been” is not a comparison of the previous week’s pain with the present week, instead, it is suggestive of the level of pain throughout the previous week. However, after translation by the experts, it was observed that the true meaning of this item had been lost, and it had to be modified after some discussion. Similarly, the word ‘thick’ in the item “I have thick mucous/phlegm” was translated using two different words. One of those words is not used commonly in the northern regions of the Karnataka state and if this questionnaire were to be administered to a person from the northern part of the state, he/she would find it inappropriate. So, the translators involved in the committee decided to avoid such terms and proceed with more widely accepted terms throughout the state.

In this study, cancer of the buccal mucosa seemed to be the most prominent primary location of the tumour. This has especially been very commonly seen among the people of South India, which is significantly associated with the increasing habit of betel quid chewing. Betel quid contains tobacco which is quite often the reason for the later development of buccal mucosa cancer [23]. The number of male participants in this study is 6 times that of the female participants, which is concurrent to the global dominances of HNC in men. A similar trend is reported in a recent study involving head and neck cancer patients from India [24].

Due to low socioeconomic status and lack of education, most patients ignore the early signs and symptoms of cancer and seek help when the symptoms become exaggerated or unmanageable. In India, majority of the patients with cancer present with advanced stage disease [25, 26]. Almost 41.6% of patients in this study were observed to have stage IV cancer. As the cancer becomes more progressive, combined modality treatment most often becomes a necessity [27]. For all operable patients with resectable locally advanced oral cavity malignancies, surgery followed by adjuvant RT with or without CT remains the gold standard of treatment care [28].

Cronbach's alpha values were greater than 0.90 in five symptom clusters and greater than 0.70 in the remaining clusters in the original version of VHNSS, indicating strong internal consistency [22]. In the Brazilian Portuguese version, Cronbach's alpha values for the domains of “swallowing solids, dry mouth, mouth pain, mucus, voice, pain, and taste/smell” ranged from “0.86 to 0.74”, while values for the domains of “nutrition, swallowing liquids, and teeth” were “0.62, 0.62, and 0.67”, respectively [29]. The Italian version exhibited good content validity while the Chinese version exhibited good internal consistency with Cronbach’s alpha values ranging from “0.74 to 0.95” [30, 31]. The translated Kannada version also showed similar findings with the overall value of α = 0.93, which means that the internal consistency of the questionnaire is excellent. The items also showed good content validity. The test–retest score obtained fell under the category of ‘good reliability’.

As all the participants in this study were head and neck cancer patients undergoing radiation therapy, they were likely to develop toxicities over the course of their treatment lasting about 6 to 8 weeks. Therefore, we carried out test retest after a period of only one week anticipating a less volatile clinical manifestation of the toxicities.

The main limitation of the study is the reduced sample size; the current study was a time bound study and we aimed to understand if the Kannada language version is a linguistically equivalent counterpart of the original version, hence the sample size was calculated based on the high prevalence HNC cancer and near certain possibility of oral toxicities in patients receiving radiation therapy. Future studies with larger samples would perhaps facilitate further reinforcing of the findings.

Another limitation of the study is that construct validity could not be assessed due to the lack of a gold standard test that measures the correlation between similar domains as that of VHNSS 2.0. Colloquial variations present within the Kannada language as well as the differences between spoken language and written language proves to be another limitation. Future scope of work can include validation of the questionnaire on survivors with long term toxicities.

## Conclusion

Sociocultural adaptation and translation of original VHNSS 2.0 into the Indian language Kannada has been successfully carried out in this study. This version can, hence, be considered a useful patient-reported tool to evaluate the oral symptomatology in HNC patients. This tool can serve as a valuable addition to the clinical speech and swallow assessment battery for HNC patients. It would also be beneficial to multiple professionals working with Kannada-speaking HNC patients. This developed version now becomes the first translated tool in the Indian regional language, Kannada, that assesses multiple domains related to oral functioning in HNC patients during or after radiotherapy.

## Data Availability

The data that support this study will be shared on reasonable request to the corresponding author.
